# Surgery

**Published:** 1879-12

**Authors:** 


### SURGERY.

INDICATIONS AND CONTRA-INDICATIONS FOR OPERATIONS ON SUBJECTS AFFECTED WITH CONSTITUTIONAL DISEASES.—M. Verneuil. (*La France Méd.*, Sept. 27.)

The following is the author's *résumé* of a paper read at the 6th meeting of the International Congress of Medical Sciences at Amsterdam.

1st. Surgical operations are not formally contra-indicated in subjects suffering from constitutional diseases; they are even under these circumstances often permitted, frequently useful, and sometimes indispensible.

2nd. Their prognosis is always much more serious than in healthy individuals; it is more uncertain, more difficult to establish, nothing being able to make us judge in advance whether the influence will be good or bad, which the operation will exercise on the general disease, any more than the manner in which this disease will react upon the local reparative process.

3rd. The prognosis differs moreover, according to the different constitutional diseases, and to the individual cases; it varies further, according to the form which it affects, the degree, and principally according to the alterations, more or less extensive and profound, undergone by the organic humors and solids.

4th. The danger inherent to the diathesis is inconsiderable when the latter is still in the state of dyscrasia, it increases notably when appreciable chemical or histological lesions appear; it becomes extreme when the large viscera, liver, kidneys, spleen, heart, lungs, present advanced lesions, as sclerosis, steatosis, amylosis, phlogosis; or when they are invaded by pathological products, special to certain diathetic states as tubercles, gummata, carcinoma, and divers neoplasms.

5th. Diathetic subjects should not be deprived of the benefits of surgical intervention, even when it is perilous. The physician should endeavor to attenuate the gravity of the prognosis, and to assure the success of the operation. He will attain this often by bringing to bear the greatest care in choosing the most proper time, the best method, the most efficacious dressing, and above all by instituting during and after the operation, and even before, if possible, a hygienic, dietetic and pharmaceutic treatment, specially adapted to the constitutional malady.

6th. To establish solidly the indications and contra-indications for operations, to calculate with some precision the chances of success or otherwise, to decide whether to operate or not, the physician should understand thoroughly the pathogeny, the pathology, the natural evolution, the terminations, and the medical treatment of the constitutional disease. This knowledge, which all surgeons do not possess sufficiently, perhaps, prevents more bloody operations than it incites, and gives greater confidence than is generally had in the efforts of nature, seconded by a relatively mild therapeutic treatment.

7th. A conscientious consideration of the immediate or remote result of operations on diathetic subjects, is of a nature to dissipate many of the illusions regarding the puissance of the surgical art. It is painful to say it, but truth obliges it, complete and durable triumphs are rare. Without doubt many operative successes are obtained, but no therapeutic success. We suppress a manifestation of the diathesis or an intercurrent affection, but many times the constitutional disease gains therefrom an intensity and rapidity. Many a scrofulous or cancerous patient would live longer if they remained under medical treatment instead of passing through the hands of the operator.

8th. It is just to add that to be more often palliative than curative, the operations in question are not less sometimes of great utility; in extreme cases they may prolong life, render it less cruel, and for the patient at least leave a chance for hope. In cases less grave, and when the constitutional disease may be successfully treated, the operation favors the cure by gaining time, in suppressing an immediate cause of danger, and in leaving the field more free for medical treatment.

---

RUSH MEDICAL COLLEGE.—CHICAGO, ILL., Dec. 1, 1879.—A concours for the lectureship on gynæcology, in the spring course of lectures in this college, will be held January 6th, 1880. Applications for admission thereto received from "regular" physicians only. All who desire to compete for the position are requested to communicate with the secretary, who will assign subjects and furnish any desired information relating to the conditions of the concours. At the last spring session of lectures in this college, 148 students were enrolled. Already 113 have matriculated for attendance upon the lectures next spring.—J. H. ETHERIDGE, *Secretary*.

